# The effects of electrical stimulation on glial cell behaviour

**DOI:** 10.1186/s42490-022-00064-0

**Published:** 2022-09-03

**Authors:** Christopher T. Tsui, Preet Lal, Katelyn V. R. Fox, Matthew A. Churchward, Kathryn G. Todd

**Affiliations:** 1 Neurochemical Research Unit, Department of Psychiatry, University of Alberta, Edmonton, AB T6G 2G3 Canada; 2 Neuroscience and Mental Health Institute, University of Alberta, Edmonton, AB T6G 2E1 Canada; 3 Department of Biomedical Engineering, University of Alberta, Edmonton, AB T6G 2V2 Canada; 4 Department of Biological and Environmental Sciences, Concordia University of Edmonton, Edmonton, AB T5B 4E4 Canada

**Keywords:** Neural interface, Microglia, Astrocyte, Oligodendrocyte, Biocompatibility, Electrical stimulation

## Abstract

Neural interface devices interact with the central nervous system (CNS) to substitute for some sort of functional deficit and improve quality of life for persons with disabilities. Design of safe, biocompatible neural interface devices is a fast-emerging field of neuroscience research. Development of invasive implant materials designed to directly interface with brain or spinal cord tissue has focussed on mitigation of glial scar reactivity toward the implant itself, but little exists in the literature that directly documents the effects of electrical stimulation on glial cells. In this review, a survey of studies documenting such effects has been compiled and categorized based on the various types of stimulation paradigms used and their observed effects on glia. A hybrid neuroscience cell biology-engineering perspective is offered to highlight considerations that must be made in both disciplines in the development of a safe implant. To advance knowledge on how electrical stimulation affects glia, we also suggest experiments elucidating electrochemical reactions that may occur as a result of electrical stimulation and how such reactions may affect glia. Designing a biocompatible stimulation paradigm should be a forefront consideration in the development of a device with improved safety and longevity.

## Background

Neural interfacing is a fast-developing technology that allows external devices to communicate with the nervous system, thereby further closing the gap between man and machine [1, 2]. Neural interfacing is often discussed in the context of improving the quality of life of a person with a disability that afflicts the nervous system, restoration of function after injury, or enhancement of function. Electrical activity is measured and/or applied to facilitate communication between an external device and the organ of interest (the brain and/or spinal cord), with the goal of eliciting activity from target sets of neurons and thereby effecting a change in function or behaviour.

Neurons are one major population of cells found in the central nervous system (CNS) – the other population are glial cells. Collectively glial cells are vital to the development, growth, and security of the CNS [3–6]. Subtypes of glia, such as microglia, astrocytes, and oligodendrocytes, all have different and numerous roles that enable and enhance neuronal function, fate, and survival [7] leading to crucial impacts on cognition and behaviour.

While glial cells differ substantially from neurons in that they are not classically excitable by electrical stimulation (i.e. they do not produce action potentials), they are highly sensitive to both the direct effects of electrical stimulation on nervous tissue and to indirect effects on nearby neurons affected by stimulation. Moreover, it has been previously shown [8–13] that there exist voltage-gated ion channels on all glia, and that they are able to communicate with each other through the use of intracellular ion fluxes. Transmembrane movement of ions (e.g. Ca^2+^, Na^+^, K^+^) are commonplace across all cells of the CNS; electrical charge is carried through these ions thus making them responsible for membrane potential changes in the CNS [14].

Neurons are structurally distinct from glia – one of the most obvious differences is that neurons feature dendrites and an axon to facilitate propagation of action potentials from one cell to the next. Neurons and glia communicate with one another via release of soluble molecules and receptor-ligand interactions [15]. Microglia are vital to neuronal development, pruning, and maintaining of homeostasis [16]. They are also constantly surveillant of their environment [17]. Although microglia do not conduct action potentials as neurons do, their functions are similarly affected by membrane potentials and ion channels present on the membrane [18, 19]. By regulating the flow of ions such as K^+^, Ca^2+^, and Cl^−^ (and therefore membrane potential and intracellular ion concentrations), ion channels are key effectors of cell activities such as migration, proliferation, morphology change, and production of cytokines and reactive oxygen species [18]. Similarly, astrocytes also feature ion channels which are used to regulate flow of ions (e.g. K^+^, Na^+^, Ca^2+^) between cytosolic and extracellular spaces [20, 21]. Transient increases in calcium ion concentrations in astrocytes, for example, have been documented to have an impact at the synapse by influencing phenomena such as plasticity and release of neurotransmitters and gliotransmitters [22, 23].

There are many applications of electrical stimulation that target the nervous system. Each application differs from another in terms of the target area, intensity of stimulation, duration of stimulation, and whether the application requires the use of an invasive implant. When an invasive implant is required, for example in deep brain stimulation (DBS), it offers a more direct and focused interface with target cells and reduces the probability of unwanted diffuse stimulation of areas adjacent to the target site [24]. The major problem with this approach is the phenomenon of glial scarring [25, 26]. Microglia and astrocytes cordon off the implant/injury site and segregate it from adjacent healthy tissue. While this normal response to foreign objects can serve to mitigate the spread of damage to adjacent healthy tissue, it also prevents nearby neurons from accessing the interface site. This makes the glial scar a significant contributor to poor signal-to-noise ratios experienced by such implants and failure of the devices altogether over a longer time-course. The stretch goal for many new invasive devices involves improving the biocompatibility of the implants that are inserted into tissue – this is to improve their service life and reduce the need for any troublesome revision surgeries. Much work has been done to mitigate the impact of the glial scarring phenomenon from a materials science approach [27–30].

There remains, however, another question that must be further and more thoroughly addressed when considering the concept of biocompatibility of neural interfacing devices: *how do glial cells respond to electrical stimulation?* In the broader literature, sufficient attention is given to how neurons respond to electrical stimulation patterns and how this translates into modified function and behaviour of the subject organism, but rarely is the response of glial cells to stimulation addressed. As glial cells are the caretakers and defenders of the nervous system, they also have a major role to play in determining the fate of other cells around them following electrical stimulation.

The present review examines available literature on how exogenous electrical stimulation affects glial cells. Summaries of experiments done in vitro and in vivo are provided, with consideration of different stimulation paradigms (e.g. direct current vs. alternating current), invasive vs. non-invasive experimental methods, along with discussion of potential cellular mechanisms of the glial response to stimulation.

## Main text

### Glial cell responses to electrical stimulation

#### Non-invasive vs. Invasive electrical stimulation

The glial response to neural interfacing devices has two major elements: the cellular response to electrical stimulation, and the response to the physical presence of an implant. While some stimulation paradigms bypass implanted electrodes (e.g. epidural stimulation, a non-invasive method) the added presence of an invasive implant elicits a foreign body response orchestrated by microglia and astrocytes. This would conceivably exacerbate any tissue response to the device. There have been many studies published which focus on the effect of invasive implants on glial cell reactivity [24, 25, 31, 32], but studies that further integrate electrical stimulation into their experiments are more limited [33]. There are invasive implant studies that focus more extensively on glial cell responses to electrical stimulation and less on responses to the implant itself. Some studies have electrodes that contact cells [34] and apply electrical field stimulation to them, but data pointing towards evidence of a foreign body response is lacking. To our knowledge, it appears that there are few studies published that concurrently detail glial cell responses to both an implant as well as any applied electrical stimulation. Doing such a concurrent assessment would greatly increase the value of a study’s appraisal of a novel neural interfacing device.

There also exist invasive studies that offer insight on some fascinating ways in which glial cells respond to electrical stimulation at the cellular level [35]. Electrical stimulation can elicit calcium ion waves in glial cells; whether this includes microglia was investigated in the paper. Calcium wave generation is made possible through adenosine triphosphate (ATP) release and purinergic receptor activation. Schipke et al*.*’s experiments showed that both astrocytes and glial precursor cells participated in Ca^2+^ waves. In response to electrical stimulation-induced Ca^2+^ waves, patch clamp recordings also revealed a transient induction of an outward rectifying K^+^ current in microglia, though this was only seen in 5 out of 13 microglial cells investigated. ATP was deduced to have been released from glia to serve, in part or in whole, as a carrier for the Ca^2+^ wave. Tetrodotoxin (TTX) and Cd^2+^ were introduced into the brain slices to exclude possible neuronal contributions to the Ca^2+^ wave (e.g. generation of actional potentials and synaptic release). Though it has been suggested that ATP coming from astrocytes results in purinergic receptor activation in nearby cells which in turn leads to rising internal calcium levels in those cells [36], it is of interest to determine whether stimulation-induced increases in extracellular ATP levels would be sufficient to act as a damage-associated molecular pattern (DAMP) for microglia thus potentially triggering their activation.

In Roitbak and Fanardjian’s study, cat cortices were subjected to electrical stimulation using implanted silver wires [37]. Electrophysiology recordings of glia did not reveal spikes that were indicative of action potentials normally seen in neurons. However, when subjected to stimulation paradigms that were higher in amplitude and frequency, depolarization was observed in affected glia (though membrane voltage decay was extremely rapid). It was suggested that the glia depolarizing was largely due to potassium ion contributions – glial cell movement could be elicited through increases in extracellular concentrations of K^+^.

High frequency stimulation (HFS) is a widely documented form of DBS [23, 38] used to suppress tremors associated with Parkinson’s disease by targeting structures in the basal ganglia (thalamus, globus pallidus, subthalamic nucleus). Generally, the usage of DBS has been accepted to be a safe and effective intervention [38]. Chronic effects of stimulation on glia appear to be highly localized at the electrode-tissue interface as exemplified by the 12-month study of DBS on pigs by Orlowski et al. [39]. The effects of HFS on astrocytes have been widely discussed over the past approximately 15 years. They are highly suspected of being involved in the increased release of ATP, its downstream product adenosine, and subsequent A1 receptor activation which result in the reduction of tremors [40]. Astrocytes have also been suspected of being responsible for glutamate release through increased influx of Ca^2+^ into the cell following stimulation [41], as well as mediate extracellular concentrations of K^+^ [42]. In the case of microglia, a study by Vedam-Mai et al. [43] suggests that DBS is helpful in reducing the number of activated microglia at and around the lesion compared to microlesion and sham animals. With regards to its capacity to contribute to the inflammatory response against an implanted electrode, microglia activity at the electrode-tissue interface is also heavily dependent on purinergic signalling. A computational model reported by Silchenko and Tass [44] presents an interesting correlation between the size of a glial scar around an implant and the amount of ATP produced from device implantation and stimulation. As well, an attenuation of fractalkine signalling due to DBS was hypothesized by Chen et al. [45] to contribute to reduced levels of microglia activation. Effects on microglia density and cell size have also been documented in certain parts of the brain as a result of DBS; according to Hadar et al. [46], the introduction of an electrode into the medial prefrontal cortex results in a local increase in microglia density and cell size which was prevented by DBS. They also interestingly found that the same experiments in the nucleus accumbens produced no significant change in microglia density and cell size even after introduction of an electrode and stimulation. The study alludes to how microglia are a heterogeneous population in the CNS [47], and the way in which they behave are at least in part due to a subject’s age, area of the CNS affected, as well as the pathology in question.

Non-invasive implants also require the use of electrodes, but they are applied without penetration of CNS tissue (e.g. transcranial direct current stimulation, tDCS) and thus do not have penetrating contacts within the tissue. An in vitro model of such an approach uses bridges made of agar or salt to connect electrolyte solutions to the cultures themselves [48–50]. In a 2015 study, Pelletier et al. cultured murine N2a neuroblastoma cells, BV2 microglial cells, and C8-D1A astrocytic cells that were exposed to direct current fields through the use of agar bridges [49]. Upon being electrically stimulated, morphological changes were noted in the glial cell types – cells either oriented themselves parallel to the electric field (microglia) or were oriented perpendicular to it (astrocytes). Further to these observations, the results suggested that such electric fields were capable of affecting both microglia and astrocytes: cyclooxygenase-2 expression in microglia was upregulated after electrical stimulation and lipopolysaccharide priming, while astrocyte metabolism was increased [51]. These observations suggested an inflammatory and hypertrophic effect, respectively.

Some modalities, such as epidural electrical stimulation (EES), are somewhat intermediate in terms of procedure invasiveness [52, 53]. EES requires an implant to be surgically placed at the dorsal surface of the spinal cord, and is necessarily more invasive than applications such as tDCS, yet lacks the target specificity offered by penetrating electrodes as used in procedures such as deep brain stimulation (DBS) and intraspinal microstimulation (ISMS). Baba et al. showed that epidural electrical stimulation of the rat brain had neuroprotective outcomes following ischemic stroke [52]. Electrical stimulation resulted in less apoptotic cells as antiapoptotic cascades were activated (Pi3 kinase/Akt signalling pathway). Upregulated levels of neurotrophic factors (glial cell line-derived neurotrophic factor, brain-derived neurotrophic factor, vascular endothelial growth factor) were observed. Electrical stimulation also enhanced angiogenesis and suppressed microglia and astrocyte proliferation.

**Fig. 1:**
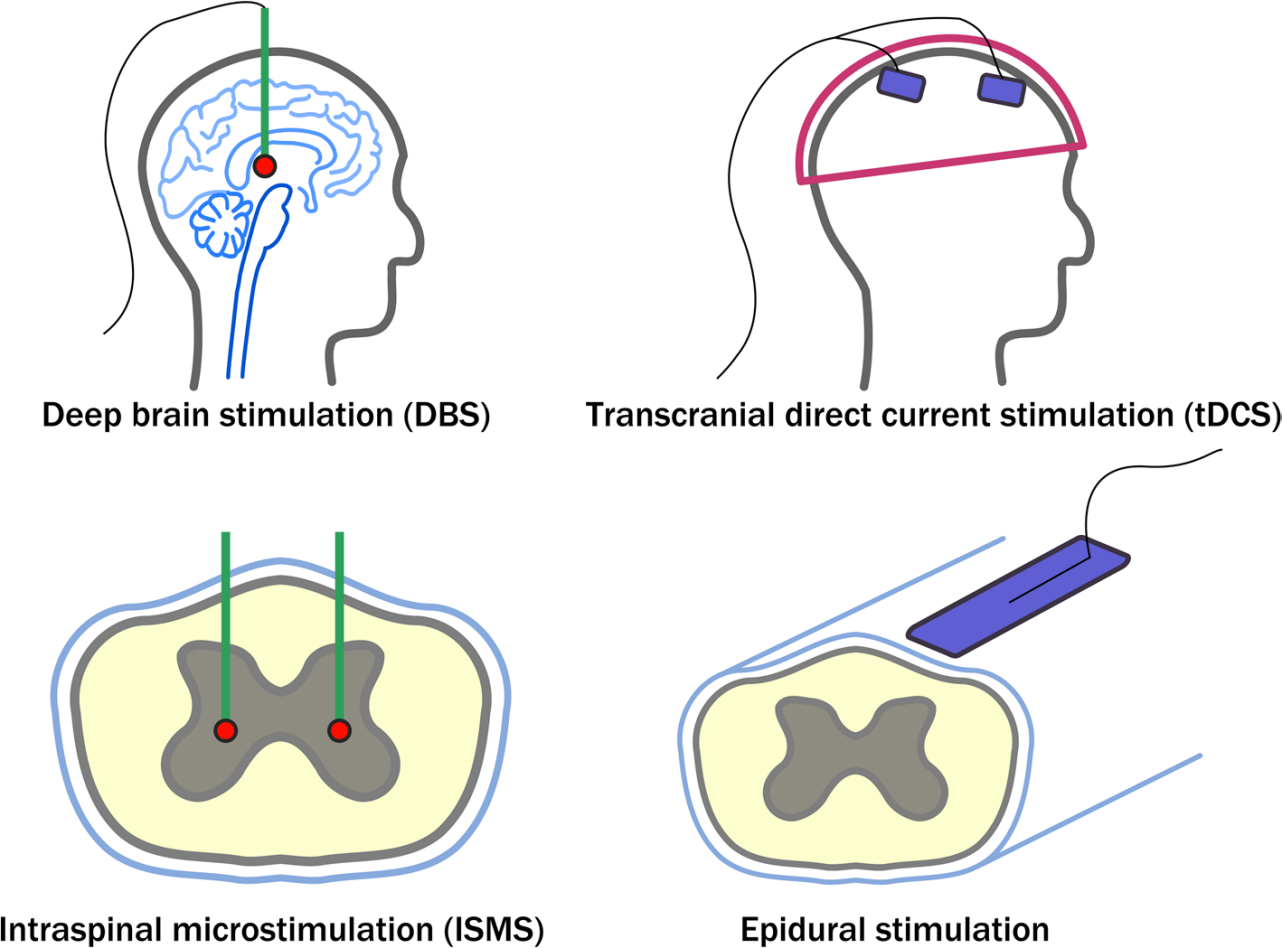
Different electrical stimulation techniques target different parts of the CNS (brain, spinal cord), and with varying levels of invasiveness

Regardless of whether stimulation utilizes an invasive implant (Fig. 1), there exists convincing evidence that electrical stimulation paradigms can manipulate glial cells in terms of their morphology and orientation, and elicit intercellular signalling among glia. It is unclear, however, if such observations translate to glia possibly taking on a more pro-inflammatory or anti-inflammatory role and how surrounding cells or tissue would be impacted by this. Further in vivo evidence suggests electrical stimulation is capable of therapeutic benefit in part by mitigating inflammation-associated proliferation of glia in the context of stroke – whether such a concept can be applied to other injuries and neurodegenerative contexts warrants further and extensive investigation.

#### Direct current vs. Alternating current

Application of electrical current to tissues is typically accomplished using either direct current (DC) and alternating current (AC). The choice of which is used for a particular stimulation paradigm depends on the application. Direct current is often used in applications such as transcranial direct current stimulation (tDCS), which (in the clinical context) makes use of electrodes placed on the outside of the head and is designed to treat disorders such as depression and Parkinson’s disease. Latchoumane et al. investigated the molecular pathways underlying the treatment effects of tDCS [54]. Embryonic stem cell-derived neuron-glia co-cultures were subjected to chronic low frequency stimulation and direct current stimulation paradigms in the presence of the excitotoxic mediator L-glutamate to simulate CNS injury. The glia in the cultures, which differentiated into O4 + oligodendrocytes and GFAP + astrocytes, upregulated transcripts for NMDA receptor subunit NR2A, brain derived neurotrophic factor (BDNF), and Ras-related protein RAB3A – collectively suggesting that electrical stimulation can modify neuronal network plasticity. A further summary of key tDCS findings is reviewed elsewhere [55]. It was shown that low intensity brief tDCS increased glucose metabolism in cultured mouse astrocytes [56], and that high intensity anodal and cathodal tDCS activated microglia [57]. In their own experiments, Gellner et al*.* exposed adult male rats to 20 min of anodal tDCS and saw morphological changes in microglia and astrocytes [55]. Their study also suggested that amoeboid microglia may be more susceptible to tDCS due to their higher abundance of voltage-gated ion channels.

Another invasive DC stimulation study utilized monophasic stimulation paradigms on rat C-fibres in the dorsal horn [58] – the mere stimulation of these fibres, even outside of any nerve damage, was sufficient to activate microglia (upregulated Iba1, IL6, etc.) and sensitize the animal to pain. DC electric fields have also been shown to serve as a helpful, instructive mechanism for neurite extension of dorsal root ganglion neurons, with electrically stimulated Schwann cells contributing heightened levels of neurotrophins [59]. It would be interesting to know if it would be possible to similarly enable axonal regeneration/neurite extension via electrically stimulated glial cells in the CNS.

In a simpler experiment, Kearns et al. showed how short-term DC stimulation of macrophage cell lines could induce expression of markers that were characteristic of M1 and M2 phenotypes [50]. M1 and M2, alternatively termed the ‘classical’ and ‘alternative’ phenotypes, respectively, describe how macrophages transition between being pro-inflammatory and anti-inflammatory. This terminology has also been applied to microglia [60, 61] and has been used in the context of other stimuli. In the context of the CNS, M1 microglia are associated with neurotoxicity and cell death, while M2 microglia are assessed to be acting in a neuroprotective role [62]. Considerable debate over the past several years suggest that microglia (and indeed peripheral macrophages) do not fit nicely into a pro-/anti-inflammatory dichotomy (or even a binary sliding scale). Rather, the way in which microglia would respond to some sort of stimulus is highly contextual; it would depend on where in the CNS the microglia are located, the nature of the stimulus/injury, how far away the microglia of interest are from the injury, and at what point during or after the injury the microglia are being observed. That said, the preceding study suggests the potential for DC stimulation to be applied to modify microglial activity to promote tissue healing.

Electrical stimulation paradigms that utilize AC feature phases of both positive and negative polarities. Such paradigms are often designed with charge balancing in mind – an opposing phase offers a way to cycle electrical charge out from any affected cells or tissue and thus avoid damage. In a recent study, Ishibashi et al. found that astrocytes *promoted* myelination in response to biphasic electrical impulses [63]. The cytokine leukemia inhibitory factor (LIF) was found to be released in larger quantities by astrocytes due to ATP release from firing axons – LIF was then found to promote myelination by mature oligodendrocytes. In another study, stimulation of C6 glioma cells using a variety of balanced and unbalanced waveforms suggested that the way in which electrical paradigms are designed had an impact on cell oxidative stress and neuroprotective behaviours [64].

Alternating current paradigms were also used to evaluate inflammation and damage in the context of electro-acupuncture stimulation of a rat Parkinson’s disease model [65]. Rats with transected medial forebrain bundles were electrically stimulated via stainless steel electrodes inserted into 2 acupuncture points: one at the head (between the ears), and another down at the cervical section of the spinal cord. Whether these electrodes made direct contact with CNS tissue is unclear. In this study, biphasic electrical stimulation protected dopaminergic neurons from microglia-mediated cytotoxic damage. It was found that survival rates of dopaminergic neurons were higher with electrical stimulation than without – this was coupled with observations that the stimulation significantly reduced TNFα and IL1β release, and that microglia activation was reduced.

Another application that utilizes charge-balanced biphasic waveforms is intraspinal microstimulation (ISMS)—a functional electrical stimulation technique that uses microwires (tens of μm in diameter) implanted into the spinal cord to elicit movement of the lower limbs following spinal cord injury. The technique has been demonstrated extensively to be effective at eliciting movements following spinal cord transections [66, 67] and is, at the time of the writing of this paper, being evaluated in clinical trials. The effects of ISMS paradigms on glial cells appear to remain limited, however. A study by Bamford et al. provides the only evidence known to the authors on this matter [33]. Microwires were surrounded by reactive astrocytes and CD68 + cells were found surrounding the microwire – this was indicative of microglia/macrophage recruitment and glial scarring. Recruitment of force was not altered upon stimulation, which suggests that not enough tissue damage was present to compromise underlying neural networks. Stimulus trains were run for 4 h/day for 30 days; further investigation into glial reactivity and force recruitment over a more chronic timecourse would help determine the maximum lifetime of that implant design in the spinal cord before device failure due to glial scarring.

Differences in cell orientation with respect to the direction of the electric field have been noted as a point of contrast between DC and AC paradigms. While orientation of glia (either parallel or perpendicular to the field) has been documented and is predictable in DC fields [49], AC stimulation had not been shown to direct orientation of migration in a consistent manner [68]. Interestingly, Ariza et al. also found that neural stem/progenitor cells (NSCs) exposed to DC field stimulation favoured differentiation into neurons rather than glia, and that AC stimulation did not favour differentiation into one cell type over another [48]. This observation would have implications in designing strategies for guiding neuronal growth/repair in a damaged nervous system.

#### In vitro and in vivo works of note

Finally, some attention should be given to the creative ways in which in vitro and in vivo electrical stimulation experiments have been designed, and the outputs that have been generated from them with respect to glial cell reactivity.

A good summary of in vitro experiments explicitly assessing glial cell responses to electrical stimulation has been compiled by Bertucci et al. [69]. Briefly, the collection of experiments characterized glial cell responses in terms of polarization towards the electrodes, cell morphologies, cell protrusion lengths, and cell body sizes. The timecourses of the experiments listed in the review provided for stimulation intervals of up to 24 h, followed by a maximum of 48 h post-stimulation monitoring. With such experiments, it would be of interest to determine glial cell responses past a 24 h time window; what, for example, would be timecourse over which glial scarring/cell death occurs in similar models? How would these phenomena change with repeated (e.g. daily) rounds of electrical stimulation applied? If these questions are addressed, any future in vitro experiments studying glial cell reactivity to electrical stimulation would better emulate chronic responses.

Cell culture systems have also been developed to study neuron-glia responses to electrical stimulation. Lee et al. utilized microfluidic systems to create spatially restricted cell cultures which then received electrical stimulation [70]. Their study remarkably showed oligodendrocytes maturing and myelinating neurons more efficiently upon exposure to an electric field. In Xu et al., cortical 3D cultures made of electrospun polypyrrole/polyacrylonitrile nanofibers were electrically stimulated [34]. The formation of cell clumps/clusters was prevented with electrical stimulation, but it did not disperse the clumps that had already formed. Electrical stimulation also increased the degree of glial cell proliferation and accelerated neuron maturation. In another study, a nanocomposite membrane comprising of poly(L-lactic-co-glycolic acid)/graphene oxide was cultured with neural stem cells as a candidate composite material for use in electrically-stimulated nerve repair [71]. The substrate improved neural stem cell proliferation and differentiation into neurons (at the expense of differentiation into astrocytes), and neurite elongation.

In spinal cord injury (SCI) in vivo studies, electrical stimulation of existing glia in the CNS results in increased GFAP expression (i.e. astrocyte hypertrophy increased) 1 week after injury [72, 73]. By preconditioning SCI rats with electrical stimulation, astrocytes were activated but secondary symptoms such as edema and necrosis were abated. Brief electrical stimulation has also been found to be beneficial for neuronal regeneration. A leech model was used to examine effects of electrical stimulation on neurons [74]. Different neurons (Retzius and P cells) responded differently to the same electrical stimulation pattern, but regardless of the pattern used more leech microglia were seen around the stimulation electrode each time which implies that neuronal regeneration is at least partly due to microglia distribution and activity.

### Device development

#### Materials and electrochemistry considerations

In addition to understanding the effects of electrical stimulation on glial cell behaviour as described in the above sections, acknowledgement must also be given to the engineering and design aspect of neural electrode implants. As far as development of invasive neural electrode implants is concerned, factors to be considered include electrode material selection, stimulation paradigms, and electrode geometry. Detailed documentation of these considerations and more can be found in a comprehensive summary by Merrill et al. [75]. Damage to neural tissue arises from mechanical (tissue/device mismatch and insertion damage) and electrochemical means.

Electrode material selection is important from a safety perspective. It goes without saying that a conducting material should be used; however, other considerations include potential material corrosion, ion leaching, degradation, and byproduct formation from electrochemical reactions. A conductive material that degrades and leaches toxic byproducts into its target environment will inevitably cause implant rejection and exacerbate inflammation and damage at the insertion site.

Common electrode materials include platinum, iridium, gold, and silicon. Carbon-based materials (e.g. graphene, carbon nanotubes) and organic materials (e.g. polyaniline, poly(3,4-ethylenedioxythiophene)) have also been more recently described in the literature [29, 31, 76–79]. Such materials are generally understood to be compatible and safe for use in CNS tissue [80]. However, the materials listed above generally will elicit a foreign body response from glial cells – that is, gliosis will ensue and a scar will form that encapsulates the implant. The extent of this response is partly dependent on the stiffness of the material – for stiffer materials such as metals, mechanical mismatch between the implants and tissue are further compounded by micromotion-induced stresses. The materials themselves are generally inert – they do not leach cytotoxic particles into the surrounding tissue by themselves. Whether electrical stimulation results in electrochemical reactions at the interface that produces cytotoxic compounds depends on the material that makes up the implant as well as the parameters of the paradigm itself [75, 81]. In the literature, common ways in which glial cell reactivity is assessed include cytokine release/expression [79], cell viability [82], morphological comparisons (e.g. ramified vs. ameboid morphologies for microglia) [83], and cell area coverage of the probe [84].

Merrill et al. [75] explains and compares different stimulation waveforms in terms of trade-offs between action potential initiation probabilities, tissue damage, and corrosion risk. Stimulation paradigm design must also take into account the target cell population. As electrode implants are primarily targeting neurons, stimulation parameters at the contact sites must be sensitive to the kinds of tissue in which the electrode is implanted – this will translate into differences in parameters such as charge per phase and pulse width [81].

Probe geometry is important, especially for implant insertion. A probe should be stiff enough to facilitate insertion into tissue, but not too stiff that tissue/device mismatch becomes problematic [85]. Alternatively and interestingly, studies have been done where novel materials such as PEDOT have been successfully polymerized in situ to form an integrated network with neural tissue [86] thus effectively blurring the border between device and tissue. The result is an electrically conductive network that is pervasive throughout local extracellular space, to the point where scar tissue can be avoided and healthy neurons can be contacted.

Electrochemical considerations are also tied to device material selection. If an electrical stimulation paradigm results in the oxidation or reduction of a chemical species, especially in a Faradaic reaction where charge is passed between electrode and electrolyte, it is desirable to add an opposing and balancing phase to reverse whatever reactions may have occurred. In addition to redox reactions involving electrode material, other chemical species in the surrounding electrolyte may also be affected by electrical stimulation. A commonly discussed theme is the need to avoid water splitting into constituent species of hydrogen and oxygen gas (i.e. keep voltages within the water window). Gas production can result in local changes in pH near the electrode and adversely affect cells [87]. In the same paper, organic compounds are also known be susceptible to redox reactions (e.g. the oxidation of glucose to gluconic acid, CO_2_). Oxygen reduction is also to be expected during stimulation pulses [88]. Reduced oxygen species can be damaging to tissue. There may be further chemical species evolved from electrical stimulation, with differences seen between in vitro (different cell culture media formulations) and in vivo (extracellular fluids) [75, 87].

## Conclusions

### Considerations for future work

**Fig. 2:**
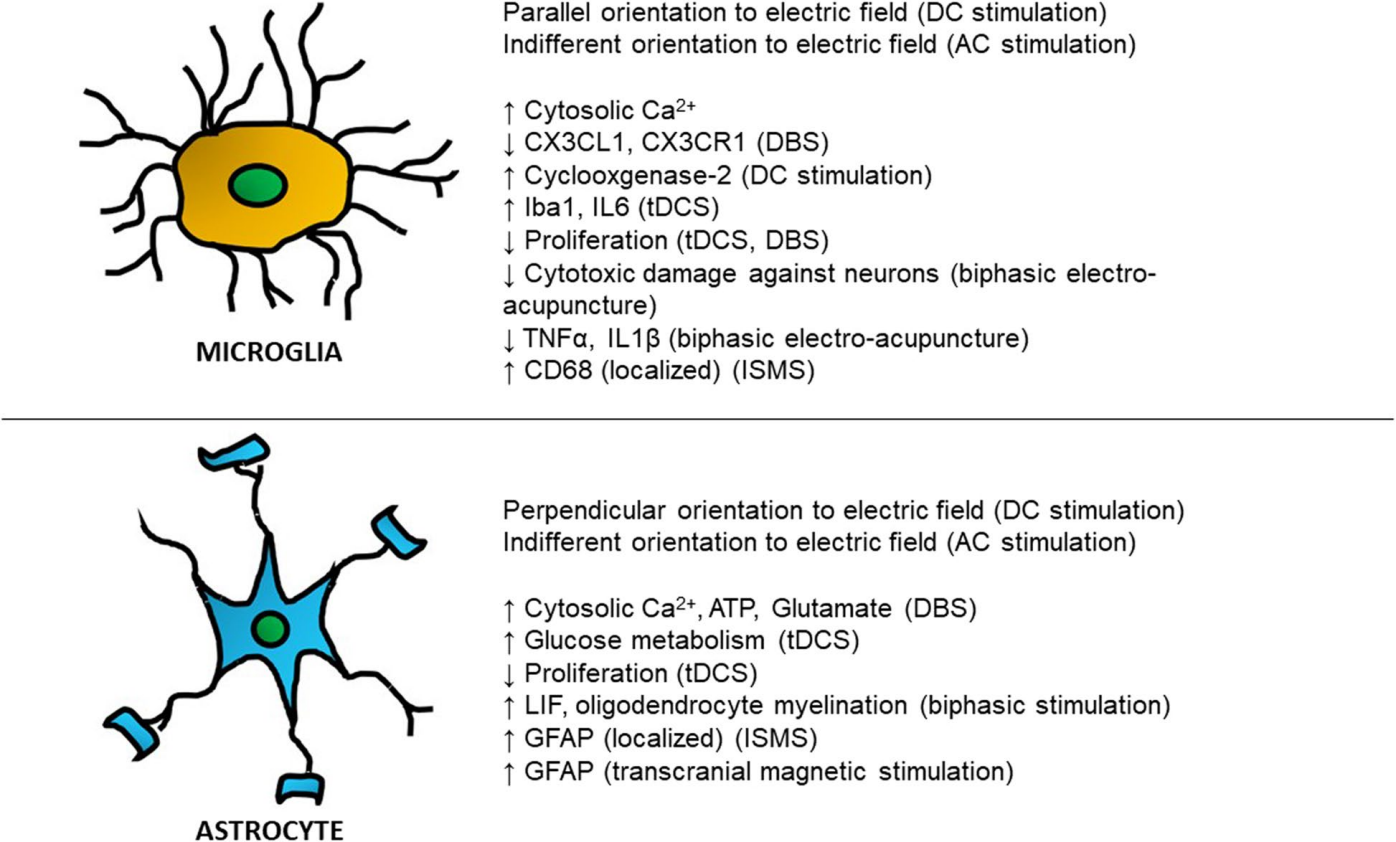
Effects of electrical stimulation differ between microglia and astrocytes, and are further complexed by different modalities and parameters of stimulation

Across the literature surveyed in this review, common themes emerge with respect to the outputs explored in the aforementioned studies (Table 1). While there are several works which suggest that electrical stimulation is foremost an inflammation-inducing action on glia, other papers utilize electrical stimulation with the perspective that it can be harnessed to promote neuroregeneration and tissue healing by using glial cells as a go-between. Caution should be exercised however – the way in which glia respond to an electrical stimulus depends very much on the nature of the stimulus itself (Fig. 2), the application, target area within the CNS, and the target cells – the full complexity of which has yet to be explored.

**Table 1:** Summary of primary studies of electrical stimulation of glia

**Study**				**Invasive / Contact with cells?**		**Current**		**Glial subtypes examined**	**Application/Purpose of study**
	**Ref**	**In vitro**	**In vivo**	**Yes**	**No**	**AC**	**DC**		
**Ariza et al., 2010**	[48]	✓			✓	✓	✓	Neural stem/ progenitor cells	Engineering of electric fields to control differentiation and growth of transplant cells
**Baba et al., 2009**	[52]		✓	Semi-invasive (Epidural)			✓	Astrocytes, Microglia	Electrical stimulation as a therapeutic treatment for cerebral ischemia
**Bamford et al., 2010**	[33]		✓	✓		✓		Astrocytes, Microglia	Intraspinal microstimulation
**Chen et al., 2020**	[45]		✓	✓			✓	Microglia	DBS suppression of fractalkine signalling in Parkinson’s rat model
**Cohen et al., 2020**	[74]		✓	✓			✓	Microglia	Electrical stimulation mediated neuronal regeneration via microglia (or via differential microglia distribution)
**Colmenárez-Raga et al., 2019**	[53]		✓	Semi-invasive (Epidural)			✓	Astrocytes, Microglia	Modulation of rat hearing sensitivity via epidural stimulation of auditory cortex
**Fu et al., 2019**	[71]	✓		✓			✓	Astrocytes	Electrical stimulation via a PLGA/graphene oxide substrate for nerve repair
**Hadar et al., 2017**	[46]		✓	✓		✓		Microglia	DBS suppression of microglia activation from perinatal CNS injury
**Hathway et al., 2009**	[58]		✓	✓			✓	Microglia	Identifying microglia role in chronic pain/central sensitization in response to C-fibre stimulation
**Ishibashi et al., 2006**	[63]	✓		✓		✓		Astrocytes, Oligodendrocytes	Electrical stimulation-induced remyelination via astrocyte activity
**Latchoumane et al., 2018**	[54]	✓		✓			✓	Astrocytes, Oligodendrocytes	Investigation into underlying molecular pathways that make tDCS work in context of CNS injury
**Lee et al., 2017**	[70]	✓		✓		✓		Oligodendrocytes	Model for studying effects of electrical stimulation on oligodendrocyte myelination activity
**Liu et al., 2004**	[65]		✓	✓		✓		Microglia	Neuroprotective role of electro-acupuncture stimulation against neurodegenerative disease
**Orlowski et al., 2017**	[39]		✓	✓		✓		Astrocytes, Microglia	Longitudinal DBS study in Goettingen pigs
**Pelletier et al., 2015**	[49]	✓			✓		✓	Astrocytes, Microglia	Identifying the mechanisms behind the clinical benefits of tDCS
**Roitbak and Fanardjian, 1981**	[37]		✓	✓			✓	Not specified	Characterization of glial cell depolarization
**Schipke et al., 2001**	[35]		✓	✓		Not specified		Astrocytes, Microglia	Proof of Ca^2+^ wave propagation through microglia using electrophysiological recordings and stimulation
**Vallejo et al., 2019**	[64]	✓			✓	✓	✓	C6 Glioma cells	Electrical stimulation-induced gene expression modulation of glia
**Vedam-Mai et al., 2016**	[43]		✓	✓		Not specified		Microglia	Assessment of extent of microglia activation following DBS
**Xu et al., 2018**	[34]	✓		✓			✓	Astrocytes, Ependymal cells	Use of electrical stimulation and nanofibers in neural tissue engineering

A great body of literature has emerged and developed over the past approximately 10 years on the biomaterial modification of invasive electrodes into neural tissue. The studies surveyed have taken a wide breadth of approaches towards mitigating the issue of glial scarring (e.g. mechanical modification of base materials, conjugation of bioactive substrates onto the electrode surface, anti-inflammatory treatments following insertion) [29]. We propose that further advancement of this field of research is required to develop more meaningful devices that could one day see clinical translation – more specifically, studies that take a biomaterials approach to modulating glial scar formation should also eventually integrate electrical stimulation into the proposed experiments. Indeed, the main function of many such devices is to deliver electrical current to tissue. It is therefore of interest to know, for example, if there are any differences in electrochemical activity around the electrode-tissue interface resulting from biomaterial modifications that could negatively impact the biocompatibility of such a device. Furthermore, would frequent electrical stimulation degrade such electrodes and cause them to weaken or fail structurally? Current insight into the range of electrochemical reactions that happen at the electrode-tissue interface is limited, but could potentially be elucidated using methodologies outlined in Cogan’s review on characterization of neural electrodes (e.g. cyclic voltammetry, electrical impedance spectroscopy, voltage transient measurements) [81]. Consider also what the threshold for stimulation-induced damage is as outlined by Shannon’s equation [89], and other aggravating factors in an organism that could contribute to the inflammatory response against an implant: implant tethering to a relatively fixed surface (e.g. skull), electrode wire micromotion, etc.

Any future in vivo and in vitro electrical stimulation studies would have added value in implementing extended time courses following stimulation (for monitoring of cell responses) and multiple rounds of stimulation into an experiment. It is anticipated that multiple rounds of stimulation will be more reflective of clinical applications where frequent (daily) usage of exogenous currents is to be expected, and that in vitro and in vivo models that show this will more accurately recapitulate any chronic cell or tissue response resulting from implant insertion and electrical stimulation.

More work also needs to be done in terms of the effects of different electrical stimulation parameters on CNS cells. As neurons come in different shapes and sizes in the CNS, designing/referencing customized paradigms for stimulating a particular group(s) of neurons in the CNS is an eventuality. What is also of interest are any changes in glial cell reactivity due to differences in stimulation parameters (e.g. AC/DC, different charge-balance schemes, current amplitude, frequency, pulse width, duty cycle, interphase delay, etc.); this is further compounded by evidence of glial cell heterogeneity throughout the CNS [47]. A more thorough understanding of the factors mentioned above will open the door to developing novel electrode and stimulation designs. This will result in reduced glial cell reactivity and translate into a longer lasting (and more effective) implant.

## Data Availability

Data sharing is not applicable to this article as no datasets were generated or analysed during the current study.

## Abbreviations

AC: Alternating current
Akt: Protein kinase B
ATP: Adenosine triphosphate
BDNF: Brain-derived neurotrophic factor
CD68: Cluster of Differentiation 68
CNS: Central nervous system
DAMP: Damage-associated molecular pattern
DBS: Deep brain stimulation
DC: Direct current
EES: Epidural electrical stimulation
GFAP: Glial fibrillary acidic protein
HFS: High frequency stimulation
Iba1: Ionized calcium binding adaptor molecule 1
IL1β: Interleukin 1β
IL6: Interleukin 6
ISMS: Intraspinal microstimulation
LIF: Leukemia inhibitory factor
NSC: Neural stem cell
PEDOT: Poly(3,4-ethylenedioxythiophene)
Pi3: Phosphatidylinositol 3
SCI: Spinal cord injury
tDCS: Transcranial direct current stimulation
TNFα: Tumor necrosis factor α
TTX: Tetrodotoxin
